# Atrial Fibrillation Modifies the Relationship Between Beta Blocker Dose and Physical Capacity After Myocardial Infarction

**DOI:** 10.3390/clinpract16040067

**Published:** 2026-03-28

**Authors:** Paulina Rabiej-Krzys, Karolina Szczygiel, Rafal Lenard, Francesco Perone, Joanna Popiolek-Kalisz

**Affiliations:** 1 Department of Cardiology, Cardinal Wyszynski Hospital in Lublin, al. Krasnicka 100, 20-718 Lublin, Poland; 2 Department of Clinical Dietetics, Medical University of Lublin, ul. Chodzki 7, 20-093 Lublin, Poland; karolina.szczygiel@umlub.pl; 3 Cardiac Rehabilitation Center, Cardinal Wyszynski Hospital in Lublin, al. Krasnicka 100, 20-718 Lublin, Poland; 4 Cardiac Rehabilitation Unit, Rehabilitation Clinic ‘Villa delle Magnolie’, 81020 Castel Morrone, Caserta, Italy; francescoperone1988@gmail.com

**Keywords:** cardiac rehabilitation, myocardial infarction, beta blocker, atrial fibrillation

## Abstract

Background: Atrial fibrillation (AF) is a common arrythmia in post-myocardial infarction (MI) cardiac rehabilitation (CR) cohorts, and beta-adrenergic signaling remodeling and rate-control pharmacotherapy may influence functional capacity. Methods: We retrospectively studied 117 consecutive male post-MI patients referred to outpatient CR. Functional capacity was assessed with a 6 min walk test (6MWT). AF was identified from clinical records, and beta-blocker exposure was unified as carvedilol-equivalent daily dose. Results: Beta-blockers were used in 94.1% of patients and AF was present in 10.3%. Patients with AF were older (72.7 ± 6.6 vs 58.1 ± 9.3 years) and walked shorter distances (430.0 [375.0–497.5] vs. 540.0 [480.0–570.0] m). In the prespecified interaction model, age remained independently associated with lower 6MWT (−4.29 m/year; *p* < 0.001), AF was associated with lower 6MWT (−137.21 m; *p* = 0.01), and the beta-blocker dose × AF interaction was positive (+6.78; *p* = 0.02; R^2^ = 0.44). Importantly, the beta-blocker dose was not associated with 6MWT in patients without AF, whereas a positive association was observed in AF (B = 7.55, *p* = 0.04). Conclusions: In this exploratory analysis, AF identified a subgroup with markedly reduced functional capacity in early post-MI CR, supporting the potential of phenotype-informed assessment. Additionally, the association between beta-blocker dose and 6MWT distance differed by rhythm status. These preliminary findings require confirmation in larger prospective cohorts.

## 1. Introduction

Cardiac rehabilitation (CR) is a structured, multidisciplinary intervention delivered to cardiovascular patients with the dual purpose of restoring functional capacity and reducing cardiovascular risk [1,2]. Beyond its established role after myocardial infarction (MI), CR is increasingly considered relevant for patients with arrhythmias, in whom symptoms and exercise intolerance results in limitations in daily functioning.

In CR studies, atrial fibrillation (AF) is frequently encountered as a comorbidity or concomitant condition that may potentially modify exercise physiology, program response, and the interpretation of functional tests. AF is a result of atrial cardiomyopathy and complex molecular remodeling that consists of inflammation, oxidative stress, fibrosis, extracellular matrix turnover, and autonomic dysregulation [3]. AF develops and persists through interacting pathways that link triggers, e.g., adrenergic and inflammatory stimuli, with a substrate characterized by structural and functional atrial remodeling [4,5,6]. This pathophysiological background is important in terms of CR because exercise training and secondary prevention interventions may potentially impact these pathways, including autonomic balance and cardiometabolic factors, while pharmacotherapy used in post-MI care (e.g., beta-blockade) directly modifies chronotropic response and may alter symptom-limited functional performance [7,8,9].

In the post-MI settings, increased sympathetic activity and β-adrenergic receptor (beta-AR) pathway dysregulation can contribute to improper excitation–contraction combination, myocardial remodeling, and arrhythmia vulnerability, which provides a rationale to examine how beta-blocker exposure impacts functional capacity during CR [10]. Beyond heart-rate lowering, beta-blockers can differ in receptor selectivity and in downstream signaling effects that may be potentially relevant to symptoms and exercise tolerance [11,12,13]. In experimental models, carvedilol has been reported to exhibit beta-1-adrenoceptor beta-arrestin-biased signaling, whereas such signaling profiles may differ for other agents commonly used after MI [14,15].

The evidence for exercise-based CR (ExCR) in patients with AF has expanded over last years. An updated Cochrane review evaluated ExCR compared with non-exercise controls in adults with any AF subtype or post-treatment for AF [16]. Earlier conclusions highlighted that, due to the limited number of randomized participants and outcome events, the impact of ExCR on mortality or serious adverse events could not be reliably evaluated, whereas pooled analyses suggested improvement in surrogate measures of physical exercise capacity, although uncertainty regarding effect magnitude due to low-to-very-low certainty evidence persisted [17]. Moreover, a recent systematic review and meta-analysis, which incorporated trial sequential analysis, reported that ExCR improved exercise capacity and AF symptoms, while no significant differences were demonstrated for mortality and serious adverse events in the available trials [18]. A further systematic review focusing on rehabilitation and AF similarly concluded that structured exercise is associated with improvements in cardiorespiratory fitness and quality of life outcomes [5]. Other overviews have also underlined that CR is feasible and generally safe in AF populations, but robust evidence for hard endpoints in AF (e.g., cardiovascular or all-cause mortality, recurrent events, or rehospitalization) remains limited [19,20,21].

Despite these advances, two clinically important gaps remain directly relevant to post-MI CR practice. First, AF may influence functional capacity during CR and may constrain the achievable response during CR through rate irregularity, impaired chronotropic competence, and reduced stroke volume reserve, particularly in patients with concomitant ventricular dysfunction. Second, the interaction between AF status and pharmacotherapy intensity, especially beta-blocker dosing, which may be escalated for rate control, may confound or modify observed functional capacity, independently of underlying cardiac structure. What is more, these issues are seldom addressed in real-world cohorts that include simultaneous echocardiographic assessment and heart-rhythm monitoring. In available studies, ectopy and arrhythmias have been evaluated during exercise testing and associated with subsequent outcomes, supporting the concept that arrhythmic burden is not merely an incidental finding but may reflect clinically important electrophysiological vulnerability [22]. Moreover, other physiological parameters beyond simple heart rate measures may carry information regarding exercise limitation in AF. For example, a recent study has assessed breathing frequency in AF patients undergoing ExCR, reflecting the broader potential of integrative physiological phenotyping within CR settings [23]. Similarly, cardiometabolic tissue compartments such as epicardial adipose tissue have been highlighted as potentially relevant contributors to AF-related remodeling and inflammation, supporting the potential of structured lifestyle and exercise interventions for impacting AF through adiposity- and inflammation-related mechanisms [24]. The increasing feasibility of home-based and technology-supported CR models, including tele-monitored programs, proves the potential implementation context in which rhythm- and pharmacotherapy-informed personalization of CR may become increasingly important [25,26].

The present study aimed to characterize determinants of functional capacity among post-MI male patients undergoing CR, with a particular focus on AF status, beta-blocker treatment, along with rhythm-monitoring parameters. We aimed to analyze if rhythm status and pharmacotherapy intensity are associated with functional capacity assessed with the 6 min walk test (6MWT).

## 2. Materials and Methods

This retrospective observational study included consecutive male patients after recent MI (in sub-acute phase) referred to a structured, outpatient early cardiac rehabilitation (CR) program. Clinical and program-related data were extracted from medical records. Functional capacity was assessed with 6MWT. AF status was based on documented diagnosis (ICD-10 code I48) and/or rhythm assessment available in the clinical records. Pharmacotherapy was focused on beta-blocker exposure and included information about the substance and dose. Then, for the purpose of the analyses, the doses of each agent were standardized by converting prescribed beta-blockers to the equivalent daily dose of carvedilol using a prespecified conversion scheme [27]. They were based on the type on substance and dose (x mg drug: y mg carvedilol) with the proportion of 1:5 for bisoprolol, 1:5 for nebivolol, 5:1 for metoprolol tartrate, and 4:1 for metoprolol succinate. The standardized equivalent dose was analyzed as a continuous variable. Because medication intensity may reflect clinical indication and comorbidity burden, selected concomitant therapies were also captured and compared between rhythm strata., i.e., use of sodium–glucose cotransporter-2 (SGLT2) inhibitors and mineralocorticoid receptor antagonists (MRA) was summarized as binary variables. Echocardiography variables (including left ventricular ejection fraction (LVEF), left ventricular end-diastolic diameter (LVEDd), diastolic interventricular septal thickness (IVSd), diastolic posterior wall thickness (LPWd), mitral inflow E velocity (E-wave), mitral inflow A velocity (A-wave), as available) and ambulatory ECG monitoring (Holter) selected indices (minimum, maximum and mean heart rate, and ventricular ectopy burden recorded as VE) were also collected. Basic clinically relevant data, such as sex, age, body mass and height, were also collected. Body mass index (BMI) was calculated as the proportion of height square expressed in meters to body mass expressed in kilograms. For subgroup analyses, men were additionally stratified by AF status.

6MWT was used as a standardized measure of submaximal functional capacity and was performed as part of routine clinical assessment at the CR center. The test was conducted in accordance with established methodological recommendations, using a flat, straight indoor corridor with a clearly marked course and turning points. Participants were instructed to walk as far as possible within 6 min at a self-selected pace, with the option to slow down or stop and resume walking if needed. Standardized instructions and encouragement were provided, and the primary outcome was the total distance walked, recorded in meters at the end of the 6 min period.

Statistical analyses were performed in R Studio (version 4.4.2, R Foundation for Statistical Computing, Vienna, Austria). For continuous variables, normality of the distribution was checked with Shapiro–Wilk test. Variables with normal distributions are presented as mean ± SD and were compared between patients with and without AF using Welch’s two-sample *t*-test. Variables that did not meet normality assumptions are presented as median [IQR] and were compared using the Wilcoxon rank-sum test (Mann–Whitney U). Categorical variables are presented as *n* (%) and were compared using Fisher’s exact test (or χ^2^ test when appropriate). Continuous variables were modeled using ordinary least squares linear regression. Univariable models were fitted with 6MWT as the outcome and each prespecified predictor was entered individually (age, BMI, AF, LVEF, IVSd, LVEDd, LVPWd, E-wave, A-wave, beta-blocker dose, and Holter indices) in the overall cohort and separately within AF strata. Then, multivariable models were fitted to evaluate independent associations and effect heterogeneity. Moreover, the primary prespecified model tested effect modification by AF status using an interaction term. Sensitivity multivariable models additionally included selected echocardiographic and Holter parameters (e.g., EF, LVEDd, VE). All models used complete-case analysis per model (patients with missing values in any variable included in a given model were excluded from that model). Two-sided *p*-values were reported, with *p* < 0.05 considered statistically significant.

## 3. Results

A cohort of 117 male post-MI patients entering outpatient CR was analyzed. Among them, 6MWT results were available in 68 patients, and that group was then included in the analysis. Importantly, included and non-included patients did not differ in terms of AF prevalence, beta-blocker dose or EF, as shown in Supplementary Table S1. Beta-blockers were used in 94.1% of the patients, while paroxysmal AF was diagnosed in 10.3% (*n* = 7), which was presented in Supplementary Figure S1. There were no patients with permanent AF. Basic characteristics of the study group is presented in Table 1. The comparison between patients with and without AF, showed significant differences in age (72.7 ± 6.6 vs. 58.1 ± 9.3 years, *p* < 0.001) and 6MWT distance (430.0 [375.0; 497.5] vs. 540.0 [480.0; 570.0] m, *p* = 0.007). The other parameters, including pharmacotherapy, did not differ between groups. The details are presented in Table 1.

In univariable linear regression analyses, age was strongly and consistently associated with lower 6MWT distance (B = −4.71 m/year, 95% CI −6.46 to −2.96; *p* = 0.000001). AF was associated with a marked reduction in 6MWT distance (B = −89.98 m, 95% CI −158.06 to −21.90; *p* = 0.010). Among echocardiographic parameters, LVEDd was inversely associated with 6MWT (B = −35.08 m per cm, 95% CI −64.72 to −5.44; *p* = 0.021), while LVEF showed only a non-significant positive trend (B = +1.65 m per %, 95% CI −0.22 to +3.52; *p* = 0.082). Regarding Holter-derived variables, ventricular ectopy burden was inversely associated with 6MWT (B = −0.029 per beat, 95% CI −0.051 to −0.007; *p* = 0.01). On the other hand, BMI, the beta-blocker dose, and Holter heart-rate metrics were not significantly associated with 6MWT in the overall group. The details are presented in Table 2.

However, when patients were stratified by AF status, the pattern in those without AF remained dominated by non-arrhythmic correlates of baseline 6MWT distance. Age retained a strong inverse association with 6MWT (B = −4.19 m/year, 95% CI −6.32 to −2.06; *p* = 0.00022;). LVEDd remained inversely associated (B = −42.25 m per cm, 95% CI −72.12 to −12.38; *p* = 0.0064), and VE remained a strong inverse correlate (B = −0.035 per beat, 95% CI −0.053 to −0.017; *p* = 0.00024). In terms of echocardiographic findings, A-wave was also inversely associated with 6MWT (B = −0.99 per cm/s, 95% CI −1.92 to −0.06; *p* = 0.037). In contrast, in patients with AF, a significant positive association between the beta-blocker dose and 6MWT was observed (B = +7.55 m per mg of carvedilol equivalent, 95% CI +0.49 to +14.61; *p* = 0.040), whereas other echocardiographic and Holter indices were not consistently associated and were characterized by wide confidence intervals. The details of the results are presented in Table 3.

Then, in the prespecified multivariable model assessing effect modification by AF status beta-blocker dose × AF) in men, age remained independently associated with lower 6MWT distance (estimate −4.29 m/year, *p* = 0.0000018). AF was associated with substantially lower 6MWT (AF estimate −137.21 m, *p* = 0.012, interpreted at the reference beta-blocker dose). Importantly, there was statistically significant effect modification by AF, i.e., the dose × AF interaction term was positive (estimate +6.78, *p* = 0.0197), indicating that the association between the beta-blocker dose and 6MWT differed by rhythm status. In that model, among patients without AF, beta-blocker dose was not associated with 6MWT (B = −0.34, *p* = 0.618), whereas among men with AF, the implied association was positive (B = +6.45 per unit, calculated as −0.34 + 6.78). This interaction model explained a moderate proportion of variance in baseline 6MWT (adjusted R^2^ = 0.44; overall model *p* < 0.000001).

The subsequent models performed separately in patients with and without AF confirmed this observation. Given the very small AF subgroup, multivariable modeling within the AF subgroup was intentionally restricted to a single model including only age and the beta-blocker dose, to reduce overfitting. More complex multivariable models were performed only in the non-AF subgroup and overall group, where the sample size was substantially larger and model stability was more acceptable. The study reflects a real-world early CR cohort, in which functional assessments are performed as part of routine care and may not be uniformly documented. The details of the models are presented in Table 4.

## 4. Discussion

In this real-life cohort of male patients entering early post-MI CR, functional capacity assessed by 6MWT was associated with age and rhythm phenotype. Exploratory analyses showed that patients with AF presented markedly lower 6MWT distance compared with those in sinus rhythm, indicating that AF can potentially identify a clinically vulnerable subgroup. While this observation is consistent with prior reports that AF is associated with reduced exercise tolerance [28], the present data additionally emphasize the practical importance of rhythm status when interpreting functional tests performed after MI. These findings also align with the broader AF–rehabilitation literature, suggesting that structured ExCR can be implemented in AF populations and may impact exercise capacity and related outcomes, while acknowledging the heterogeneity in program content, intensity prescription, and outcomes, as well as the limitations of the randomized evidence base [16,16,29]. In this context, the large performance gap observed in patients with AF underscores the clinical importance of rhythm phenotyping at CR entry and supports the hypothesis that AF can be a potential modifier of functional status in post-MI secondary prevention pathways.

A key observation in the present preliminary analysis was association modification by AF status in the relationship between the beta-blocker dose and 6MWT distance. In the interaction model, the association between the beta-blocker dose and 6MWT differed by rhythm status: among patients without AF, the beta-blocker dose was not associated with 6MWT, whereas among men with AF, higher beta-blocker dose correlated with higher 6MWT. Because the AF subgroup was small, these analyses are presented as preliminary rather than confirmatory evidence of effect modification. We nevertheless report these preliminary signals to indicate the need for prospective studies with systematic capture of AF status and functional assessments. The real-world nature of the cohort improves external validity but also contributes to non-standardized assessment pathways and missing data. A potential explanation of the observed trend in AF is that functional capacity assessed with 6MWT may be more sensitive to pharmacologically modifiable rate-control and adrenergic pathways than in sinus rhythm, where non-arrhythmic determinants such as age may dominate. On the other hand, it is important to note that the beta-blocker dose may be potentially influenced by the symptom burden, clinician preference, hemodynamic tolerance, concomitant heart failure, and other unmeasured factors; however, in routine practice, particularly when arrhythmia is present, dose escalation is most often driven by the need to achieve adequate ventricular rate control, within the limits imposed by blood pressure and tolerability. Nevertheless, the interaction results suggest a possible phenotype-specific association between rate-control pharmacotherapy intensity and 6MWT distance that warrants further investigation.

In the cardiovascular system, beta-adrenergic receptors regulate chronotropy, inotropy and lusitropy. After MI, sustained sympathetic activation and adaptive changes in adrenergic signaling may contribute to myocardial remodeling and altered exercise response. In AF, autonomic imbalance and adrenergic surges can modulate atrial excitability and ventricular rate, thus rate-control strategies commonly rely on beta-blocker titration. In this exploratory study, to enable quantitative comparison across different beta-blockers prescribed in routine care (metoprolol succinate, bisoprolol, nebivolol), we standardized daily doses using a prespecified carvedilol-equivalent conversion scheme. Importantly, no participant received carvedilol itself, so the ‘carvedilol-equivalent’ variable should be interpreted solely as a harmonized dose metric and not as reflecting carvedilol-specific pharmacodynamic effects. It should also be noted that the relatively modest beta-blocker doses observed in this cohort should be interpreted in the context of early post-MI care. As the cohort consisted of patients in the subacute post-MI phase entering early CR, prescribed doses may in many cases reflect ongoing clinical titration and hemodynamic tolerability rather than stable long-term target dosing. On the other hand, the mean heart rate on Holter monitoring was approximately 63 beats/min, indicating that satisfactory rate control had already been achieved in many patients, which may also partly explain the absence of further dose escalation.

In patients without AF, the inverse association between ventricular ectopy burden and 6MWT suggests that electrical instability in post-MI myocardium may reflect a broader pathophysiological load that limits submaximal performance during CR. However, VE is a marker rather than a mechanistic measurement, and its association with functional capacity may reflect multiple upstream processes (autonomic imbalance, scar-related substrate, residual ischemia, ventricular remodeling) that cannot be disentangled within the current study.

The present findings refer to AF as sustained by interacting pathways involving inflammation, oxidative stress, fibrosis, extracellular matrix turnover, and autonomic dysregulation. Available evidence emphasize that these pathways contribute both to AF maintenance and to systemic phenotypes that potentially influence exercise tolerance and CR response [2]. Although biomarker measurements were not available in the present cohort, these models provide a biological explanation for why AF status may impose a functional limitation during CR and why pharmacological modulation of adrenergic signaling could potentially show rhythm-dependent associations with observed functional performance assessed with 6MWT.

Notably, in the AF subgroup, we observed a positive association between the beta-blocker dose and 6MWT distance, while the Holter mean heart rate did not differ between AF and non-AF groups and was not a significant correlate of 6MWT in the overall cohort. This pattern suggests that the relationship between the beta-blocker dose and functional performance in AF may not be explained solely by between-group differences in average heart rate. However, due to the observational design, small AF subgroup, and the use of heterogeneous beta-blockers, these findings should be regarded as preliminary and further hypothesis-generating and do not imply that higher beta-blocker doses improve functional capacity. Therefore, the observed association may reflect phenotype-related treatment role rather than a direct dose-dependent functional effect.

On the other hand, several limitations of this study should be considered. First, the study is observational and retrospective; therefore, causal inference regarding medication effects is not possible. A key limitation is that a substantial proportion of baseline 6MWT results was unavailable due to non-uniform routine documentation and the use of alternative functional assessments in clinical practice. Although patients with and without baseline 6MWT documentation were comparable in AF prevalence, beta-blocker equivalent dose, and key clinical variables, residual selection bias cannot be fully excluded and the mechanism of missingness cannot be definitively classified in this retrospective study. Moreover, missing data for echocardiography and Holter monitoring resulted in reduced effective sample sizes in multivariable models, which may introduce selection and limit precision. What is more, the AF subgroup itself was small, increasing the risk of unstable estimates which enabled only basic models to avoid overfitting.

The study cohort was heterogeneous with respect to LVEF, which should be acknowledged when interpreting the findings, particularly because guideline-based indications and therapeutic priorities for beta-blocker therapy may differ across LVEF phenotypes [30]. However, patients with and without AF did not differ in LVEF in the present cohort, suggesting that differences in ventricular systolic function are unlikely to explain the observed rhythm-specific pattern in the association between beta-blocker dose and 6MWT distance. Nevertheless, the sample size was insufficient for additional LVEF-subgroup analyses, so larger studies are needed to determine whether the relationship between beta-blocker dose and exercise capacity assessed with 6MWT differs according to both rhythm status and LVEF profile.

Male-only design is another limitation of the study. Women are underrepresented among patients entering early CR [31], and based on our preliminary assessments inclusion of women would have produced very small sex-specific AF subgroups. Moreover, as AF pathophysiology, clinical presentation, and rate-control responses can potentially differ by sex, these findings should not be extrapolated to women or to the broader post-MI population. Larger prospective cohorts with adequate female representation and systematic capture of the AF phenotype (burden, symptom severity) are needed to validate and extend these observations.

## 5. Conclusions

Exploratory analyses in this real-world cohort suggested that among post-MI men entering CR, AF is associated with shorter 6MWT distance. Further subgroup analyses suggested that the relationship between the beta-blocker dose and 6MWT performance may differ by rhythm status. However, given the small AF subgroup, these observations should be interpreted as preliminary. Our findings support the consideration of incorporating rhythm status into early CR assessment alongside standard structural and clinical evaluation, while the observed beta-blocker-related pattern requires external confirmation. Larger prospective studies with systematic functional testing are required to confirm these associations and to determine whether AF-related functional limitation is modifiable through individualized rate-control strategies and tailored CR pathways.

## Figures and Tables

**Table 1:** Basic characteristics of the study group and comparison between patients with and without AF.

Parameter [Unit]	Overall (*n* = 68)	Patients Without AF (*n* = 61)	Patients with AF (*n* = 7)	*p*-Value
Age [years]	59.6 ± 10.1	58.1 ± 9.3	72.7 ± 6.6	<0.001
Body mass index [kg/m^2^]	27.9 [26.0; 30.4]	28.5 ± 4.0	29.4 ± 5.7	0.668
Beta-blocker equivalent dose [mg]	12.50 [12.50; 25.00]	12.50 [12.50; 25.00]	12.50 [6.25; 25.00]	0.745
Any beta-blocker [%]	94.1%	93.4%	100.0%	1.000
Bisoprolol use (%)	39.7%	37.7%	57.1%	0.423
Bisoprolol daily dose [mg]	5.00 [2.50; 5.00]	5.0 [2.5; 5.0]	5.0 [4.4; 5.0]	0.857
Nebivolol use [%]	39.7%	42.6%	14.3%	0.230
Nebivolol daily dose [mg]	2.50 [2.50; 5.00]	3.8 [2.5; 5.0]	2.5 [2.5; 2.5]	0.584
Metoprolol succinate use [%]	14.7%	13.1%	28.6%	0.272
Metoprolol succinate daily dose [mg]	25.00 [25.00; 50.00]	37.5 [25.0; 50.0]	25.0 [25.0; 25.0]	0.296
SGLT-2 inhibitors use	57.4%	57.4%	57.1%	1.000
Mineralocorticoid receptor antagonist use	69.1%	72.1%	42.9%	0.190
NT-proBNP [pg/mL]	450.5 [104.8; 1003.5]	339.5 [100.0; 985.0]	499.0 [463.0; 2927.0]	0.483
LVEF [%]	50.0 [40.0; 55.0]	50.0 [43.8; 55.0]	51.0 [38.5; 57.5]	0.782
IVSd [cm]	1.1 [1.0; 1.2]	1.1 [1.0; 1.2]	1.1 [1.0; 1.1]	0.940
LVEDd [cm]	5.1 ± 0.5	5.1 ± 0.5	5.0 ± 0.3	0.538
LVPWd [cm]	1.0 [0.9; 1.1]	1.0 [0.9; 1.1]	1.0 [0.9; 1.1]	0.919
Mitral inflow E velocity [cm/s]	59.5 [50.2; 75.5]	59.0 [51.0; 71.0]	80.0 [41.0; 87.0]	0.502
Mitral inflow A velocity [cm/s]	71.2 ± 19.9	71.4 ± 17.4	69.2 ± 40.5	0.911
Holter HR minimum [bpm]	45.0 [42.0; 48.5]	45.0 [43.0; 49.0]	43.5 [40.5; 46.5]	0.418
Holter HR maximum [bpm]	107.0 [97.0; 117.0]	108.0 [97.0; 117.0]	102.5 [93.2; 106.5]	0.325
Holter mean HR [bpm]	63.0 [59.5; 67.0]	63.0 [60.0; 67.0]	63.5 [54.0; 67.8]	0.690
Ventricular ectopy burden [*n*]	11.0 [3.0; 97.0]	11.0 [3.0; 103.0]	4.0 [2.2; 36.5]	0.467
6MWT distance [m]	535.0 [470.0; 570.0]	540.0 [480.0; 570.0]	430.0 [375.0; 497.5]	0.007

AF—atrial fibrillation, 6MWT—six-minute walk test, LVEF—left ventricular ejection fraction, LVEDd—left ventricular end-diastolic diameter, IVSd—diastolic interventricular septal thickness, LPWd—diastolic posterior wall thickness, SGLT-2—sodium–glucose cotransporter-2, HR—heart rate.

**Table 2:** Univariable predictors of 6MWT distance in the overall group.

Parameter [Unit]	B	95% CI	*p*-Value
Age [years]	−4.706	−6.457 to −2.955	<0.001
Body mass index [kg/m^2^]	−3.361	−8.795 to 2.073	0.221
AF [occurrence]	−89.977	−158.057 to −21.896	0.010
LVEF [%]	1.65	−0.216 to 3.516	0.082
IVSd [cm]	87.48	−18.003 to 192.962	0.102
LVEDd [cm]	−35.076	−64.716 to −5.435	0.021
LVPWd [cm]	68.974	−66.285 to 204.234	0.312
Mitral inflow E velocity [cm/s]	0.159	−1.723 to 2.041	0.866
Mitral inflow A velocity [cm/s]	−0.499	−1.879 to 0.882	0.472
Beta-blocker dose [mg equivalents]	0.843	−1.239 to 2.924	0.422
Holter HR minimum [bpm]	−0.955	−3.658 to 1.748	0.482
Holter HR maximum [bpm]	−0.412	−1.483 to 0.659	0.445
Holter mean HR [bpm]	−0.709	−4.06 to 2.643	0.674
Ventricular ectopy burden [*n*]	−0.029	−0.051 to −0.007	0.010

AF—atrial fibrillation, 6MWT—six-minute walk test, LVEF—left ventricular ejection fraction, LVEDd—left ventricular end-diastolic diameter, IVSd—diastolic interventricular septal thickness, LPWd—diastolic posterior wall thickness, HR—heart rate.

**Table 3:** Univariable predictors of 6MWT distance in the AF subgroups.

Parameter	Without AF			With AF		
	B	95% CI	*p*-Value	B	95% CI	*p*-Value
Age [years]	−4.191	−6.321 to −2.060	<0.001	−7.522	−15.26 to 0.215	0.055
Body mass index [kg/m^2^]	−4.077	−10.165 to 2.012	0.185	2.943	−15.032 to 20.919	0.691
LVEF [%]	1.951	−0.122 to 4.024	0.065	−0.708	−8.655 to 7.239	0.828
IVSd [cm]	89.496	−18.214 to 197.206	0.101	−133.333	−3505.642 to 3238.975	0.918
LVEDd [cm]	−42.251	−72.118 to −12.384	0.006	73.232	−619.581 to 766.046	0.784
LVPWd [cm]	71.491	−35.81 to 178.792	0.187	6.098	−2058.577 to 2070.772	0.994
Mitral inflow E velocity [cm/s]	−0.06	−2.163 to 2.043	0.955	2.543	−1.886 to 6.971	0.165
Mitral inflow A velocity [cm/s]	−0.991	−1.918 to −0.064	0.037	0.455	−7.431 to 8.341	0.866
Beta-blocker dose [mg equivalents]	0.327	−1.814 to 2.467	0.761	7.551	0.488 to 14.615	0.040
Holter HR minimum [bpm]	−1.67	−4.372 to 1.031	0.22	0.345	−29.794 to 30.485	0.976
Holter HR maximum [bpm]	−0.552	−1.682 to 0.578	0.332	−0.291	−12.331 to 11.749	0.950
Holter mean HR [bpm]	−1.583	−4.735 to 1.568	0.318	0.884	−20.337 to 22.105	0.913
Ventricular ectopy burden [*n*]	−0.035	−0.053 to −0.017	<0.001	0.115	−5.355 to 5.585	0.956

AF—atrial fibrillation, 6MWT—six-minute walk test, LVEF—left ventricular ejection fraction, LVEDd—left ventricular end-diastolic diameter, IVSd—diastolic interventricular septal thickness, LPWd—diastolic posterior wall thickness, HR—heart rate.

**Table 4:** Multivariable models of the 6MWT distance in the overall group and selected subgroups.

Group	Predictor	B	*p*-Value for Predictor	R^2^	*p*-Value for Model
Overall	Age [years]	−3.064	<0.001	0.481	<0.001
	Beta-blocker dose [mg equivalents]	0.122	0.878		
	LVEF [%]	1.480	0.170		
	LVEDd [cm]	−10.164	0.609		
	Mitral inflow A velocity [cm/s]	−0.068	0.870		
	AF status	−92.976	0.054		
	Ventricular ectopy burden [*n*]	−0.024	0.125		
Without AF	Age [years]	−3.965	<0.001	0.431	<0.001
	Beta-blocker dose [mg equivalents]	−0.031	0.970		
	LVEF [%]	1.668	0.135		
	LVEDd [cm]	−6.178	0.762		
	Mitral inflow A velocity [cm/s]	0.013	0.976		
	Ventricular ectopy burden [*n*]	−0.023	0.162		
Without AF	Age [years]	−3.578	<0.001	0.323	<0.001
	Ventricular ectopy burden [*n*]	−0.033	0.060		
	LVEF [%]	1.475	0.111		
Without AF	Age [years]	−3.954	<0.001	0.314	<0.001
	Ventricular ectopy burden (VE) [*n*]	−0.033	0.054		
With AF	Age [years]	−4.634	0.188	0.789	0.040
	Beta-blocker dose [mg equivalents]	6.358	0.045		
Overall	Age [years]	−4.286	<0.001	0.443	<0.001
	Beta-blocker dose [mg equivalents]	−0.335	0.618		
	AF status	−137.208	0.012		
	Beta-blocker dose: AF status	6.783	0.020		

AF—atrial fibrillation, 6MWT—six-minute walk test, LVEF—left ventricular ejection fraction, LVEDd—left ventricular end-diastolic diameter, HR—heart rate.

## Data Availability

The original contributions presented in this study are included in the article/Supplementary Material. Further inquiries can be directed to the corresponding author.

## Supplementary Materials

The following supporting information can be downloaded at https://www.mdpi.com/article/10.3390/clinpract16040067/s1, Figure S1: Flowchart of patients’ enrollment; Table S1: Comparison of patients included and excluded from the analysis.
