# Supplementary material for: Atrial Fibrillation Modifies the Relationship Between Beta Blocker Dose and Physical Capacity After Myocardial Infarction
Source: Clin Pract. 2026 Mar 28;16(4):67. doi: 10.3390/clinpract16040067 (PMC13115022; doi:10.3390/clinpract16040067)

Supplementary Table S1. Comparison of patients included and excluded from the analysis.

**Variable**	**Included**	**Excluded**	***p***
Beta-blocker dose	5.00 [2.50; 6.25]	5.00 [1.88; 10.00]	0.884
Ejection fraction	50.00 [40.00; 55.00]	48.00 [40.00; 55.00]	0.255
Age	59.50 [51.50; 66.00]	65.00 [56.00; 71.00]	0.0384
Holter mean HR	63.00 [59.00; 67.00]	67.00 [61.00; 73.00]	0.0306
BMI	27.91 [26.03; 30.42]	28.74 [24.84; 32.08]	0.792
VEBs	11.00 [3.00; 103.00]	9.00 [2.00; 216.00]	0.997
Atrial fibrillation occurence	7 (10.3%)	7 (14.3%)	0.571

Supplementary Figure S1. Flowchart of patients' enrollment.

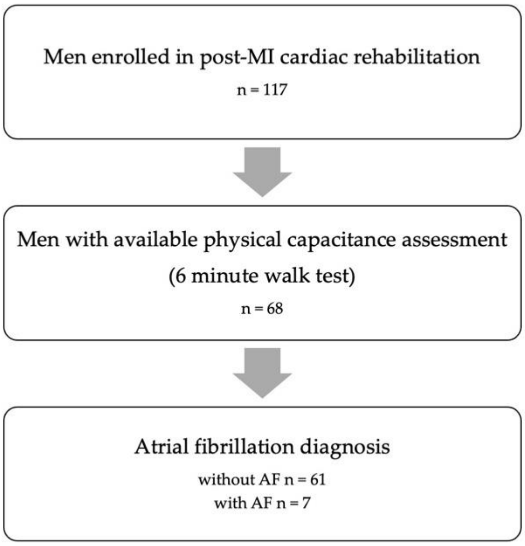

Supplement: Supplementary file 1 [file clinpract-16-00067-s001.zip › clinpract-4164269-supplementary.pdf]
